# P195: Orthopedic surgical infections caused by rapidly growing mycobacteria: integrative review of literature

**DOI:** 10.1186/2047-2994-2-S1-P195

**Published:** 2013-06-20

**Authors:** RNT Turrini, MPF Azevedo, RA Lacerda

**Affiliations:** 1 Medical Surgical Department, Nursing School of São Paulo University, São Paulo, Brazil

## Introduction

Infections due to rapidly growing mycobacteria (RGM) are strongly related to failures in the processes of cleaning, disinfection and sterilization of medical products.

## Objectives

The objective of this study was to analyze the occurrence of surgical site infections by RGM in patients undergoing orthopedic procedures.

## Methods

The method was the integrative review.

## Results

The 21 articles reported 34 cases. The median time of diagnosis of SSI was 80 days, interquartile range 352 days and mode of 90 days. The most prevalent signs and symptoms reported by patients were: pain (61.8%), secretion (50.0%), edema (41.2%), fever (41.2%), erythema (26.5%), fistula (20.6%), heat (14.7%), tremor (5.9%), abscess (5.9%) and hematoma (3.0%). Regarding surgical interventions performed in patients after diagnosis of SSI, the most frequent was antibiotic therapy (100%), removal of the orthopedic prosthetic device (50.0%), drainage (41.2%), surgical debridement (41.2%), irrigation (23.5%), surgical revision (17.6%), replacement of prosthetic devices (8.8%), removal of the prosthetic components (8.8%), and reimplantation of the prosthesis (2.9%). The identification of etiological agent(s) of SSI did not follow a routine methodology, which could influence the reliability of the results, especially regarding the kind of etiologic agent. The isolated RGM of the infection sites were M.fortuitum (the most prevalent), M.chelonae, M.abscessus, M.goodii, M.smegmatis, M.farciongenes and M.wolinsky. When the sensivity test was performed, it was observed that the strains has approximately 80,0% of sensitivity to amikacin, claritromycin, ciprofloxacin. Suspicious sources were hydro massage tub used by a resident surgeon before operating; liquid components or cement powder of methylmethacrylate or metal prosthesis; cortisone injections for chronic synovitis during five years before surgery; air conditioning system or soaking solution to rinse the prosthetic device; soap in the water, where it was accomplished the immersion of the foot (podiatris’ recommendations); bioabsorbable screws used in surgery; intra-articular injections of dexamethasone; however, none of them could be confirmed.

## Conclusion

M. fortuitum was the RGM most frequent, some infections were diagnosed after one year and it contradicts CDC’ definition of surgical site defined by CDC. The authors of the studies analyzed didn’t follow a methodological description what compromised the conclusions.

## Disclosure of interest

None declared

